# Determinants of agreement between self-reported and parent-assessed quality of life for children in Germany-results of the German Health Interview and Examination Survey for Children and Adolescents (KiGGS)

**DOI:** 10.1186/1477-7525-9-102

**Published:** 2011-11-23

**Authors:** Ute Ellert, Ulrike Ravens-Sieberer, Michael Erhart, Bärbel-Maria Kurth

**Affiliations:** 1 Department of Health Reporting, Robert Koch Institute, Seestr. 10, 13353 Berlin, Germany; 2 Child Public Health, Department of Child and Adolescent Psychiatry, Psychotherapy and Psychosomatics, University Medical Centre Hamburg-Eppendorf, Martinistr. 52, 20246 Hamburg, Germany

## Abstract

**Background:**

The aim of this study is to quantify the level of agreement between self-reporting and proxy-assessment of children's health-related quality of life using KINDL-R in a large population based study in Germany and to identify factors which are associated with agreement.

**Methods:**

The German Health Interview and Examination Survey for Children and Adolescents included the KINDL-R questionnaire on health-related quality of life. 6388 children and adolescents filled in the questionnaire while their parents answered the proxy version. Means and standard deviation for the self- and proxy ratings, and also the Pearson und Intra-Class correlation coefficients for the absolute agreement were calculated. The relationship between other variables and parent-child agreement were determined by means of logistic regression.

**Results:**

In the 'Physical', 'Self-esteem' and 'School' dimension and for the 'Total' score, the parents significantly overestimated the quality of life of their child. In contrast, the quality of life of the children in the dimensions 'Psychological well-being' and 'Family' were considerably underestimated by the parents. The proportion of parent-child ratings in agreement (difference < 0.5 standard deviations) ranges from 34.9% for the 'Self-esteem' scale to 51.9% in the 'Psychological' scale. The most important factor explaining parents rating was the level of the child's self-assessment followed by the parent's assessment of the subjective health, or reported emotional abnormalities.

**Conclusions:**

Our study shows that parental reports cannot adequately replace self-assessment for 11-17 year olds. In view of the different underlying perspectives, the parental assessments should where possible only be regarded as providing supplementary information.

## Background

In recent years, increasing importance has been attached to health-related quality of life (HRQoL) in child and adolescent medicine. The measurement of HRQoL of children and adolescents is meanwhile at least as important as for adults in clinical and public health studies [1]. The focus of interest is on the perception and evaluation of an individual's own life from a subjective perspective. For this reason, self-reporting is generally preferable to proxy assessments. However, this is only possible for children and adolescents who are capable of providing the necessary information as a result of their age, their cognitive development, and their state of health.

Solans et al. [2] identified 30 generic and 64 disease-specific instruments to register the quality of life of children and adolescents. Some generic as well as some disease-specific instruments draw only on the self-reporting of the children and adolescents. A number of instruments (43% of generic instruments and 30% of disease-specific ones) have versions both for parental (proxy) assessment and self-assessment. Some methods are based solely on information provided by parents.

There is considerable disagreement about the value of external assessments (by teachers, experts, parents). It has been argued that children/adolescents may operate within different reference systems and thus differ from adults in their understanding of HRQoL [3]. While parents can easily identify behavioural problems, this may not be the case with emotional problems such as sadness or tension [4]. Parents often lack first-hand information, for example, regarding the school experience or the social interactions of their children with friends. On the other hand, parent proxy reports could be also regarded as providing important complementary information about children's QoL [5]. It has been argued that discrepancies between self and proxy reports could validly reflect each respondent's perspective and not merely inaccuracy or bias [6].

A number of studies and reviews in recent years have compared self-assessment and information provided by proxy [3]. Whereas parents as a rule overestimate their healthy child's health-related quality of life [7-10], parents of chronically ill children tend to rate their health-related quality of life lower than the children do themselves. This has been shown for children with cerebral palsy [11] and for children with cancer [10,12]. In contrast, Chang et al. found that parents overestimated the health-related quality of life of their children with cancer [13,14].

Various factors influence the extent of agreement or difference between the assessments of parents and children, they differ depending on the direction of the deviation and they affect different dimensions of the quality of life [11,13,15]. The level of child/parent agreement also depends on the level of the quality of life [7,15].

In a study of 500 children with cerebral palsy aged 8 to 12 years in seven European countries, White-Koning et al. [11] found that high levels of parental stress were more likely to be associated with an overestimation of the child's quality of life, whereas parents were likely to underestimate the quality of life of children with severe pain. In some studies, associations were found between the self-assessments of the quality of life and the sex [7,13] or the age of the children [7,9,11,13], and between proxy assessments and the age of the parents [12] or their level of education [11,12]. Intercultural differences were found in a Europe-wide study in the extent of the agreement between assessments by proxies and children [15].

As part of the German Health Interview and Examination Survey for Children and Adolescents (KiGGS) of the Robert Koch Institute, the children self-report- and the parent proxy-report version of the KINDL-R quality of life instrument was employed. The psychometric properties of both versions had been examined and reported in a companion paper [16]. Overall both versions were found to enable a reliable and valid assessment of children's quality of life. However some differences were seen [16]. The aim of the present paper thus is to further examine the origin of these differences. Our first aim here is to quantify the level of agreement between children self-report and parent proxy reported quality of life. Second, we want to identify sociodemografic-, socioeconomic- and health-status-factors which are associated with a better or poorer agreement.

## Methods

### Design and sample

The German Health Interview and Examination Survey for Children and Adolescents (KiGGS) was carried out by the Robert Koch Institute from 2003 to 2006. Details of the preparation and implementation of this health survey are described elsewhere [17-20]. The survey involved a total of 17 641 children and adolescents aged 0-17 years. There was a 66.6% rate of participation. Since key socio-demographic and health-related characteristics for children and parents could be registered for two-thirds of the non-respondents, basic information is available for 89% of the target population and could be compared between responders and non-responders. The response analyses are described in detail elsewhere [18,21]. The study was approved by the Charité-Universitätsmedizin Berlin ethics committee and the Federal Office for the Protection of Data.

For this evaluation, we used data from 6 388 children and adolescents aged 11-17 years with complete parent-child pairs, because only for the 11-17 years olds, the KINDL-R questionnaire is presented in parallel for self-reporting and proxy assessment.

### The KINDL-R

KiGGS included the KINDL-R questionnaire on health-related quality of life [22], which has previously been tested psychometrically and clinically in epidemiological investigations as a quality of life instrument [16,23-25]. In contrast to most quality of life instruments for minors, which had originally been developed in English and then translated into German in a methodologically laborious process, the revised KINDL-R questionnaire is a German-language instrument which can be used with both clinical populations and also healthy children and adolescents. The KINDL-R is a questionnaire with 24 items, covering the following six dimensions of the quality of life over the past week: 'Physical', 'Psychological', 'Self-esteem', 'Family', 'Friends' and 'School', available in 23 languages. The time needed to complete differs between 5 and 15 minutes according child's age. The mean time is 15 minutes.

Both a self-assessment KINDL-R questionnaire and a proxy version (accompanying parents or caregivers) are available. Answers can be given in five categories (never, seldom, sometimes, often, always). It is possible to calculate a 'Total' score for the health-related quality of life from all 24 items. All measurements are scored on a scale from 0-100 points, and the higher the value then the better the quality of life. For the 11-17 years olds, the KINDL-R questionnaire is presented in parallel for self-reporting and proxy assessment. Norms for Germany are available in [26].

### Associated factors

Factors which could potentially be influential were: age and sex of child, the proxy (mother, father, mother and father, or another person), region of residence (former East or West Germany), migration background, social status of the family, child rated family climate, indications of mental abnormalities by means of the Strengths and Difficulties Questionnaire SDQ (normal, borderline, abnormal), the parental assessment of the child's state of health (very good/good, medium, or poor/very poor), a need for increased care as assessed by the parent according to the screener for children with special health care needs (CSHCN) (yes/no), any pain in the last three month, and the value of the self-assessed quality of life.

Information on covariates was obtained from self-administered questionnaires from parents and also from the children themselves (in children aged 11 years and older). The 10 federal states of the Federal Republic of Germany before reunification were defined as West Germany, whereas the five new federal states covering the region of the former German Democratic Republic and the federal state of Berlin were defined as East Germany. Data on parents' income, occupational status, and educational and occupational qualification from the parental questionnaire were used to quantify the socio-economic status (SES) of the children and adolescents as low, middle or high. Each of the three components was rated with a point system (1-7 points). The sum was calculated and categorised into the following groups: (1) low SES (3-8 points); (2) medium SES (9-14 points); and (3) high SES (15-21 points) [20]. Participants were referred to as migrants if they had immigrated themselves and had at least one parent who was not born in Germany or was of non-German nationality, or if both parents had immigrated or were of non-German nationality [27]. Family protecting factors were obtained using a shortened form of nine items of the family climate scale [28]. The parents filled in a questionnaire including a screening measure of emotional and behavioural problems in their children (Strengths and Difficulties Questionnaire SDQ). The SDQ contains 25 items assessing internalising and externalising problems on four subscales (emotional problems, behavioural problems, inattention/hyperactivity, peer problems) and, as strengths, prosocial behaviour on one subscale. The four problem subscales are summed up to a total difficulties score [29]. The CSHCN screener includes five items each subsuming one or two filter questions. The items refer to ''use of prescribed medicine'', ''above average use of or need for medical, mental, or educational services'', ''functional limitations in comparison to other children of same age'', ''use of or need of special therapies'' and ''treatment or counselling for emotional or developmental problems'' [30]. Information on pain in the last three month was obtained from the children themselves [31].

### Statistical analysis

The statistical evaluation was carried out using SPSS Version 14.0. In order to take account of the grouped data structure the 95% confidence interval were determined with the SPSS-14 procedure for complex samples. Weighting factors were introduced to correct for unequal sampling probabilities and to ensure that the survey population was representative of the national child population.

Agreement was evaluated at the individual level as well as at the group level. For each quality of life dimension, we calculated the mean and standard deviation for the self- and proxy ratings, and also the Pearson und Intra-Class correlation coefficients for the absolute agreement. The mean difference (child value minus parent value) was determined and standardised by dividing the value by the mean standard deviation of both scores (effect size) [32], thus the direction of disagreement between the self- and proxy ratings could be specified. As an additional indicator of agreement, the mean of the absolute value of the difference between values for children and parents was determined [33].

Since self-report questionnaires are regarded as the primary method of assessing HRQoL, the self-assessment was arbitrarily set as the reference point. Similar to other studies [11,34,35] and according to the usual definition of a clinically important difference in the health-related quality of life, self-assessment and parental assessment were rated as "in agreement" when the absolute difference was less than or equal to half the standard deviation of the child's values [36]. This distribution based method was also recommended in [37]. The standard deviation of the child-self report was used since this was comparable to the standard deviation in parent's/proxies' data (with higher children's SD in most scales), our definition of agreement sufficiently regards the variability of both respondent's scores. Of the cases which were not in agreement, we distinguished between those where the parents gave a lower estimate of the quality of life of their child (underestimation: parent < child) and those where the parents gave a higher estimate of the quality of life of their child (overestimation: parent > child).

The relationship between other variables (associated factors) and parent-child agreement were determined by means of logistic regression.

## Results

### Sample characteristics

A total of 6388 parent-child pairs were available for the analysis. The answers to the questionnaire were provided by the mother in a large majority of cases (83.5%). About a tenth of questionnaires were completed by the father, and in 5% of cases both parents responded. Further characteristics of the study population can be found in Table 1.

**Table 1:** Characteristics of the study population

	Girls		Boys		Total	
	
	N = 3293		N = 3179		N = 6472	
	%	95%-CI	%	95%-CI	%	95%-CI
Age group (in years)						
11-13	**39.9**	(38.9-40.8)	**39.9**	(38.9-40.9)	**39.9**	(39.0-40.8)
14-17	**60.1**	(59.2-61.1)	**60.1**	(59.1-61.1)	**60.1**	(59.2-61.0)
Respondent						
mother	**85.2**	(83.4-86.7)	**82.0**	(80.3-83.5)	**83.5**	(82.3-84.7)
father	**9.2**	(8.0-10.7)	**12.4**	(11.0-13.9)	**10.8**	(9.9-11.9)
mother and father	**4.6**	(3.8-5.6)	**4.9**	(4.1-5.7)	**4.8**	(4.2-5.4)
Region of residence						
East	**18.6**	(14.0-24.3)	**18.5**	(13.9-24.1)	**18.5**	(14.0-24.2)
Migrant background						
Yes	**15.2**	(13.3-17.4)	**15.1**	(13.0-17.4)	**15.2**	(13.3-17.4)
Social status						
Low	**26.9**	(25.0-28.8)	**26.9**	(24.9-28.9)	**26.0**	(24.0-28.0)
Intermediate	**47.7**	(45.6-49.9)	**47.2**	(45.2-49.2)	**25.4**	(23.4-27.6)
Upper	**47.5**	(46.0-48.9)	**26.0**	(24.0-28.0)	**25.7**	(24.0-27.4)
Family Climate						
Borderline	**8.2**	(7.5-9.0)	**6.9**	(6.0-7.9)	**9.6**	(8.5-10.8)
In deficit	**12.3**	(11.4-13.3)	**12.0**	(10.8-13.3)	**12.6**	(11.3-14.1)
Parent rated total SDQ						
Borderline	**5.5**	(4.5-6.6)	**7.8**	(6.7-9.1)	**6.7**	(5.9-7.5)
Abnormal	**5.4**	(4.5-6.4)	**8.5**	(7.4-9.7)	**7.0**	(6.3-7.7)
Parent rated health status						
Very good/good	**92.2**	(91.0-93.2)	**91.6**	(90.4-92.6)	**91.9**	(91.1-92.6)
moderate	**7.5**	(6.5-8.6)	**8.1**	(7.1-9.3)	**7.8**	(7.1-8.6)
Bad/very bad	**0.4**	(0.2-0.8)	**0.3**	(0.2-0.7)	**0.3**	(0.2-0.6)
CSHCN-Screener						
Positive	**14.7**	(13.2-16.4)	**16.6**	(15.4-18.0)	**15.7**	(14.7-16.7)
Pain (last 3 month)						
Yes	**77.5**	(76.3-78.6)	**71.6**	(69.8-73.2)	**83.6**	(82.1-85.1)

CI: confidence interval

### Self-proxy agreement

In three of the six quality of life dimensions ('Physical', 'Self-esteem', 'School') and for the 'Total' score, the parents significantly overestimated the quality of life of their child (Table 2). In contrast, the quality of life of the children and adolescents in the dimensions 'Psychological well-being' and 'Family' were considerably underestimated by the parents. 'Friends' is the only dimension for which the parental assessment switches with age between too low and too high. Here the parents gave the 11-13 year-olds a lower quality of life whereas for the 14-17 year-olds they reported a higher quality of life.

**Table 2:** Comparison of Means of Child and Parent Reports

		Children		Parents		Correlation		Difference		abs. Difference	
	N	Mean	95%CI	Mean	95%CI	Pearson	ICC	MW	Effect size	MW	SD
All											
Total	6,388	**72.6**	(72.4-72.9)	**74.3**	(74.0-74.6)	0.49	0.49	-1.6	0.16	8.2	6.5
Physical	6,277	**70.7**	(70.2-71.2)	**74.1**	(73.5-74.6)	0.46	0.45	-3.4	0.20	13.8	11.4
Psychological	6,318	**81.1**	(80.7-81.5)	**79.3**	(78.9-79.7)	0.32	0.32	1.9	0.14	11.5	10.2
Self-Esteem	6,332	**58.4**	(57.9-58.8)	**67.4**	(66.9-67.8)	0.27	0.23	-9.0	0.54	17.3	14.1
Family	6,302	**81.9**	(81.5-82.3)	**76.4**	(76.0-76.8)	0.47	0.44	5.5	0.36	12.9	10.6
Friends	6,386	**77.5**	(77.0-77.9)	**77.2**	(76.8-77.6)	0.41	0.41	0.3	0.02	11.9	10.2
School	6,182	**66.7**	(66.1-67.3)	**71.4**	(70.9-72.0)	0.47	0.45	-4.7	0.29	13.5	11.3
Girls											
Total	3,141	**71.3**	(70.8-71.7)	**73.9**	(73.5-74.3)	0.51	0.49	-2.6	0.25	8.4	6.7
Physical	3,089	**67.1**	(66.3-67.9)	**71.6**	(70.9-72.4)	0.48	0.46	-4.5	0.27	14.3	11.6
Psychological	3,112	**80.3**	(79.7-80.9)	**79.0**	(78.4-79.6)	0.34	0.34	1.3	0.10	11.7	10.3
Self-Esteem	3,119	**56.1**	(55.4-56.8)	**67.2**	(66.6-67.8)	0.30	0.30	-11.1	0.66	17.7	14.5
Family	3,108	**81.4**	(80.8-82.0)	**76.5**	(75.9-77.2)	0.48	0.45	4.9	0.31	12.9	10.6
Friends	3,139	**76.6**	(76.1-77.2)	**76.7**	(76.2-77.2)	0.43	0.43	-0.1	0.00	11.8	10.0
School	3,059	**66.3**	(65.6-67.1)	**72.4**	(71.6-73.1)	0.50	0.50	-6.0	0.36	13.6	11.3
Boys											
Total	3,247	**74.0**	(73.6-74.4)	**74.7**	(74.3-75.1)	0.48	0.48	-0.7	0.07	8.0	6.3
Physical	3,188	**74.2**	(73.6-74.7)	**76.5**	(75.8-77.2)	0.41	0.40	-2.3	0.15	13.3	11.2
Psychological	3,206	**81.9**	(81.4-82.4)	**79.5**	(79.0-80.0)	0.29	0.29	2.4	0.19	11.3	10.1
Self-Esteem	3,213	**60.6**	(59.9-61.3)	**67.5**	(66.9-68.1)	0.25	0.23	-6.9	0.41	16.9	13.6
Family	3,194	**82.4**	(81.9-83.0)	**76.3**	(75.7-76.9)	0.47	0.43	6.2	0.41	12.9	10.6
Friends	3,247	**78.3**	(77.7-78.9)	**77.7**	(77.2-78.2)	0.40	0.40	0.6	0.04	12.0	10.3
School	3,123	**67.0**	(66.3-67.8)	**70.5**	(69.9-71.2)	0.44	0.43	-3.5	0.21	13.5	11.3
11 to 13 years											
Total	2,901	**74.6**	(74.1-75.1)	**75.2**	(74.7-75.6)	0.43	0.43	-0.6	0.06	8.2	6.4
Physical	2,864	**74.2**	(73.5-74.8)	**75.4**	(74.6-76.2)	0.43	0.42	-1.2	0.08	13.2	11.0
Psychological	2,870	**82.8**	(82.3-83.3)	**79.4**	(78.8-80.0)	0.26	0.25	3.4	0.28	11.5	10.0
Self-Esteem	2,883	**56.2**	(55.3-57.0)	**67.6**	(66.9-68.2)	0.21	0.17	-11.4	0.69	18.9	14.5
Family	2,869	**83.9**	(83.3-84.5)	**76.5**	(75.8-77.1)	0.39	0.34	7.4	0.53	13.4	10.8
Friends	2,901	**80.3**	(79.6-81.0)	**76.9**	(76.3-77.5)	0.38	0.37	3.4	0.24	12.1	10.5
School	2,810	**70.9**	(70.0-71.8)	**75.1**	(74.4-75.9)	0.41	0.40	-4.2	0.27	13.2	11.3
14 to 17 years											
Total	3,417	**71.4**	(71.0-71.7)	**73.7**	(73.3-74.1)	0.52	0.51	-2.4	0.22	8.3	6.6
Physical	3,413	**68.4**	(67.7-69.0)	**73.2**	(72.5-73.9)	0.48	0.46	-4.9	0.28	14.2	11.6
Psychological	3,448	**80.0**	(79.5-80.5)	**79.2**	(78.7-79.7)	0.35	0.35	0.8	0.06	11.5	10.3
Self-Esteem	3,449	**59.9**	(59.3-60.4)	**67.2**	(66.7-67.8)	0.32	0.28	-7.4	0.44	16.2	13.6
Family	3,443	**80.6**	(80.0-81.2)	**76.4**	(75.8-76.9)	0.52	0.50	4.2	0.26	12.6	10.5
Friends	3,485	**75.6**	(75.1-76.2)	**77.4**	(76.9-77.9)	0.45	0.44	-1.8	0.13	11.7	9.9
School	3,372	**63.9**	(63.2-64.6)	**69.0**	(68.3-69.6)	0.47	0.44	5.1	0.31	13.7	11.3

ICC: Intraclass Correlation coefficient

Directional difference: child score-parent score

Absolute difference: |child score-parent score|

Effect size: |Mean directional difference|/[(SD_child _- SD_paren_t)/2]

CI: confidence interval

The correlations between the values for parents and children were low to moderate (a maximum of 0.52 for Pearson and 0.51 ICC). In most quality of life dimensions the effect size of the mean difference was moderate (< 0.5). The effect size was above 0.5 for 'Self-esteem', and in the case of the 11-13 year-olds also for the 'Family' scale. The 'Self-esteem' scale also showed the greatest absolute differences.

**Figure 1:**
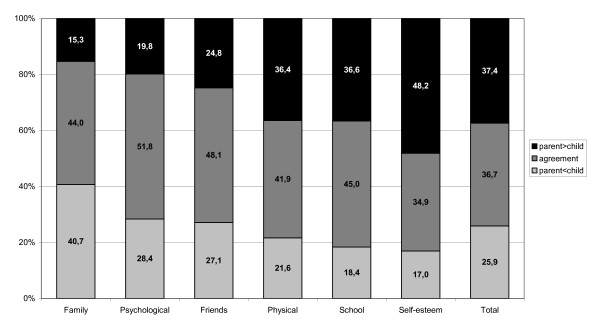
**Distribution of agreement/over- or under-estimation between child and parental reports in the dimensions of HRQoL**.

The proportion of parent-child ratings in agreement (difference < 0.5 SDs) ranges from 34.9% for the 'Self-esteem' scale to 51.8% in the 'Psychological' scale (Figure 1). For the 'Total' score, 36.7% were in agreement. For three of the six scales ('Family', 'Psychological', 'Friends'), the disagreements between parent and child were mainly due to parents overestimating their child's quality of life. This proportion was largest for the 'Family' scale, with 40.7%. In the dimensions 'Physical', 'School' and 'Self-esteem' and in the 'Total' score, the proportion of parental overestimations was larger than the parental underestimation of the quality of life of the children. The proportion of parents underestimating the quality of life of their child was smallest for 'Self-esteem' (17.0%), and in this case the proportion of overestimations was largest (48.2%).

### Multivariate analysis

Tables 3 and 4 show the result of the multivariate analysis to explain the over- or under-estimation by the parents of their child's quality of life. The higher the self-assessed quality of life (child rating), the greater is the probability that the parents will underestimate the quality of life in all dimensions (Table 3). If the parents report behavioural abnormalities of their child, then for each dimension there is an increased probability that the parents will underestimate the quality of life. The same applies if the parents report the state of health of their child not as very good.

**Table 3:** Parent < child disagreement

	Total		Physical		Psychological		Self-Esteem		Family		Friends		School	
	OR	95%CI	OR	95%CI	OR	95%CI	OR	95%CI	OR	95%CI	OR	95%CI	OR	95%CI
**Age (years)**	**1.00**	(0.96-1.05)	**1.00**	(0.96-1.04)	**0.96**	(0.92-1.00)	**1.00**	(0.95-1.05)	**0.97**	(0.93-1.00)	**0.92**	(0.89-0.96)	**1.10**	(1.05-1.15)
**Gender**														
Girls	**1**		**1**		**1**		**1**		**1**		**1**		**1**	
Boys	**1.01**	(0.87-1.17)	**0.85**	(0.72-1.00)	**0.89**	(0.77-1.02)	**1.08**	(0.90-1.30)	**1.08**	(0.93-1.24)	**0.90**	(0.77-1.05)	**1.30**	(1.09-1.56)
**respondents**														
Mother	**1**		**1**		**1**		**1**		**1**		**1**		**1**	
Father	**0.84**	(0.64-1.10)	**0.82**	(0.62-1.08)	**1.05**	(0.82-1.36)	**0.93**	(0.68-1.27)	**0.98**	(0.80-1.19)	**1.02**	(0.80-1.29)	**1.37**	(1.05-1.79)
Mother and Father	**0.60**	(0.41-0.87)	**0.89**	(0.62-1.28)	**0.72**	(0.49-1.07)	**0.50**	(0.32-0.78)	**0.82**	(0.58-1.18)	**0.51**	(0.35-0.76)	**1.04**	(0.66-1.64)
Other Person	**0.40**	(0.20-0.82)	**0.91**	(0.34-2.42)	**0.82**	(0.37-1.79)	**1.05**	(0.44-2.48)	**1.04**	(0.49-2.20)	**0.96**	(0.54-1.68)	**1.42**	(0.71-2.85)
**region**														
East	**1**		**1**		**1**		**1**		**1**		**1**		**1**	
West	**1.19**	(1.00-1.42)	**0.99**	(0.83-1.20)	**1.17**	(1.01-1.37)	**1.17**	(0.96-1.42)	**1.65**	(1.42-1.90)	**1.13**	(0.96-1.32)	**0.86**	(0.71-1.03)
**Migration background**														
No	**1**		**1**		**1**		**1**		**1**		**1**		**1**	
Yes	**1.13**	(0.87-1.47)	**1.07**	(0.83-1.39)	**0.88**	(0.69-1.11)	**1.01**	(0.71-1.45)	**0.62**	(0.50-0.77)	**0.79**	(0.59-1.05)	**1.84**	(1.42-2.39)
**Social status**														
Low	**1**		**1**		**1**		**1**		**1**		**1**		**1**	
Intermediate	**0.97**	(0.80-1.17)	**0.81**	(0.66-1.00)	**1.03**	(0.86-1.24)	**1.08**	(0.86-1.35)	**1.12**	(0.93-1.33)	**1.15**	(0.96-1.39)	**0.96**	(0.77-1.20)
Upper	**1.07**	(0.85-1.11)	**0.83**	(0.65-1.06)	**0.97**	(0.79-1.19)	**1.13**	(0.85-1.49)	**1.10**	(0.91-1.33)	**1.34**	(1.06-1.68)	**0.87**	(0.67-1.13)
**Family climate**														
Normal	**1**		**1**		**1**		**1**		**1**		**1**		**1**	
Borderline	**1.10**	(0.81-1.49)	**0.99**	(0.75-1.31)	**1.36**	(1.08-1.71)	**1.55**	(1.15-2.11)	**1.21**	(0.97-1.50)	**1.12**	(0.88-1.42)	**1.05**	(0.81-1.36)
In deficite	**1.09**	(0.78-1.53)	**0.94**	(0.66-1.34)	**1.32**	(0.95-1.83)	**0.80**	(0.54-1.19)	**1.65**	(1.24-2.20)	**1.16**	(0.85-1.58)	**0.94**	(0.64-1.40)
**Parent rated total SDQ**														
Normal	**1**		**1**		**1**		**1**		**1**		**1**		**1**	
Borderline	**4.31**	(3.15-5.91)	**1.79**	(1.37-2.34)	**3.67**	(2.67-5.05)	**3.19**	(2.22-4.56)	**3.31**	(2.47-4.44)	**2.26**	(1.65-3.10)	**2.47**	(1.75-3.49)
Abnormal	**7.56**	(5.34-10.69)	**2.10**	(1.53-2.89)	**5.31**	(3.83-7.36)	**3.75**	(2.68-5.27)	**3.64**	(2.75-4.83)	**4.69**	(3.42-6.43)	**3.84**	(2.79-5.28)
**Parent rated health status**														
Very good	**1**		**1**		**1**		**1**		**1**		**1**		**1**	
Good	**1.62**	(1.37-1.91)	**1.87**	(1.54-2.27)	**1.75**	(1.49-2.06)	**1.44**	(1.16-1.78)	**1.26**	(1.10-1.44)	**1.30**	(1.12-1.50)	**1.44**	(1.19-1.75)
Moderate/bad/very bad	**2.76**	(2.04-3.74)	**4.72**	(3.50-6.38)	**2.61**	(1.86-3.64)	**1.80**	(1.22-2.64)	**1.15**	(0.86-1.55)	**1.95**	(1.43-2.66)	**2.35**	(1.72-3.22)
**CSHCN-Screener**														
Negativ	**1**		**1**		**1**		**1**		**1**		**1**		**1**	
Positiv	**1.39**	(1.13-1.71)	**1.01**	(0.80-1.26)	**1.19**	(0.97-1.45)	**0.99**	(0.75-1.29)	**1.23**	(1.02-1.49)	**1.27**	(1.02-1.58)	**0.97**	(0.78-1.21)
**pain (last 3 month)**														
No	**1**		**1**		**1**		**1**		**1**		**1**		**1**	
Yes	**1.15**	(0.98-1.35)	**1.78**	(1.49-2.13)	**1.23**	(1.05-1.44)	**1.14**	(0.91-1.44)	**1.12**	(0.95-1.32)	**0.98**	(0.83-1.15)	**1.15**	(0.94-1.41)
**Child rating (10 points)**	**2.56**	(2.26-2.90)	**1.50**	(1.39-1.62)	**2.19**	(1.99-2.42)	**1.99**	(1.83-2.18)	**1.95**	(1.81-2.09)	**2.18**	(2.00-2.38)	**1.74**	(1.61-1.88)
R^2^	38.6		30.9		42.6		48.2		35.2		40.1		36.1	

**Table 4:** Parent > child-disagreement

	Total		Physical		Psychological		Self-Esteem		Family		Friends		School	
	OR	95%CI	OR	95%CI	OR	95%CI	OR	95%CI	OR	95%CI	OR	95%CI	OR	95%CI
**Age (years)**	**0.98**	(0.94-1.02)	**1.02**	(0.98-1.07)	**0.98**	(0.94-1.03)	**0.95**	(0.91-1.00)	**1.03**	(0.98-1.09)	**1.05**	(1.01-1.10)	**0.89**	(0.85-0.92)
**Gender**														
Girls	1		**1**		**1**		**1**		**1**		**1**		**1**	
Boys	**1.07**	(0.94-1.23)	**1.32**	(1.13-1.56)	**1.16**	(0.99-1.36)	**1.01**	(0.86-1.18)	**1.02**	(0.82-1.26)	**1.23**	(1.03-1.46)	**0.87**	(0.73-1.03)
**respondents**														
Mother	1		**1**		**1**		**1**		**1**		**1**		**1**	
Father	**1.07**	(0.85-1.34)	**1.23**	(0.97-1.56)	**1.14**	(0.87-1.48)	**1.31**	(1.03-1.67)	**1.18**	(0.87-1.60)	**0.94**	(0.73-1.21)	**1.02**	(0.80-1.31)
Mother and Father	**1.11**	(0.79-1.56)	**1.55**	(1.13-2.11)	**1.12**	(0.72-1.74)	**1.27**	(0.87-1.85)	**0.86**	(0.51-1.45)	**0.81**	(0.54-1.23)	**0.75**	(0.52-1.08)
Other Person	**0.61**	(0.31-1.17)	**2.08**	(1.12-3.88)	**0.47**	(0.11-1.94)	**0.70**	(0.29-1.71)	**1.16**	(0.44-3.03)	**0.81**	(0.38-1.72)	**0.55**	(0.27-1.09)
**region**														
East	1		**1**		**1**		**1**		**1**		**1**		**1**	
West	0.99	(0.84-1.16)	**0.99**	(0.84-1.18)	**0.90**	(0.74-1.10)	**1.10**	(0.96-1.27)	**0.91**	(0.75-1.10)	**0.98**	(0.82-1.17)	**1.23**	(1.05-1.44)
**Migration background**														
No	**1**		**1**		**1**		**1**		**1**		**1**		**1**	
Yes	**1.47**	(1.15-1.88)	**0.92**	(0.72-1.19)	**1.33**	(0.95-1.87)	**1.28**	(0.96-1.72)	**1.79**	(1.35-2.39)	**1.36**	(1.05-1.76)	**0.67**	(0.53-0.85)
**Social status**														
Low	1		**1**		**1**		**1**		**1**		**1**		**1**	
Intermediate	0.87	(0.73-1.03)	**1.00**	(0.82-1.21)	**0.75**	(0.60-0.93)	**1.04**	(0.85-1.28)	**1.01**	(0.78-1.29)	**0.81**	(0.66-0.99)	**1.03**	(0.87-1.23)
Upper	0.91	(0.75-1.11)	**1.02**	(0.83-1.25)	**0.78**	(0.61-1.00)	**1.07**	(0.87-1.33)	**0.90**	(0.67-1.19)	**0.66**	(0.53-0.83)	**1.18**	(0.96-1.46)
**Family climate**														
Normal	**1**		**1**		**1**		**1**		**1**		**1**		**1**	
Borderline	**0.86**	(0.70-1.07)	**1.16**	(0.91-1.48)	**0.71**	(0.54-0.94)	**0.98**	(0.78-1.21)	**0.84**	(0.65-1.09)	**0.85**	(0.66-1.10)	**0.97**	(0.79-1.20)
In deficite	**0.73**	(0.55-0.97)	**1.22**	(0.92-1.61)	**0.50**	(0.35-0.73)	**0.62**	(0.47-0.84)	**1.15**	(0.80-1.64)	**0.91**	(0.67-1.24)	**1.04**	(0.80-1.36)
**Parent rated total SDQ**														
Normal	**1**		**1**		**1**		**1**		**1**		**1**		**1**	
Borderline	**0.35**	(0.24-0.50)	**0.65**	(0.47-0.90)	**0.27**	(0.17-0.44)	**0.54**	(0.38-0.75)	**0.47**	(0.32-0.68)	**0.39**	(0.26-0.58)	**0.47**	(0.34-0.67)
Abnormal	**0.15**	(0.10-0.23)	**0.36**	(0.24-0.54)	**0.15**	(0.09-0.27)	**0.23**	(0.16-0.33)	**0.27**	(0.16-0.47)	**0.30**	(0.19-0.47)	**0.38**	(0.27-0.53)
**Parent rated health status**														
Very good	**1**		**1**		**1**		**1**		**1**		**1**		**1**	
Good	**0.55**	(0.47-0.65)	**0.54**	(0.45-0.64)	**0.67**	(0.55-0.79)	**0.62**	(0.52-0.74)	**0.92**	(0.74-1.13)	**0.68**	(0.58-0.81)	**0.74**	(0.64-0.87)
Moderate/bad/very bad	**0.22**	(0.15-0.31)	**0.26**	(0.18-0.37)	**0.38**	(0.23-0.62)	**0.41**	(0.29-0.57)	**0.72**	(0.48-1.10)	**0.45**	(0.32-0.65)	**0.59**	(0.43-0.82)
**CSHCN-Screener**														
Negativ	1		**1**		**1**		**1**		**1**		**1**		**1**	
Positiv	0.93	(0.75-1.16)	**0.86**	(0.69-1.09)	**0.73**	(0.53-1.02)	**0.79**	(0.64-0.98)	**0.91**	(0.72-1.15)	**0.78**	(0.59-1.02)	**0.92**	(0.74-1.14)
**pain (last 3 month)**														
No	1		**1**		**1**		**1**		**1**		**1**		**1**	
Yes	0.91	(0.76-1.10)	**0.90**	(0.75-1.08)	**1.07**	(0.85-1.34)	**0.93**	(0.79-1.10)	**0.91**	(0.72-1.15)	**1.23**	(1.01-1.51)	**1.07**	(0.88-1.28)
**Child rating (10 points)**	**0.28**	(0.25-0.32)	**0.50**	(0.47-0.53)	**0.26**	(0.23-0.29)	**0.39**	(0.36-0.42)	**0.46**	(0.43-0.50)	**0.40**	(0.37-0.43)	**0.47**	(0.44-0.50)
R^2^	38.6		30.9		42.6		48.2		35.2		40.1		36.1	

Regarding the 'Physical' domain of quality of life, the chance of parental underestimation is lower for boys than girls, whereas for the 'School' dimension it is higher for boys. With increasing age of the children, the parents are less likely to underestimate their child's quality of life with respect to Friends; in contrast, the probability of underestimating in the 'School' dimension increases with the age of the child. Parents with a migration background are more likely to underestimate the school-related quality of life of their children than parents without a migration background, whereas in the 'Family' dimension the chance of parents underestimating is lower in migrant families than families without a migration background. If mother and father respond to the questionnaire together then there is a lower change of underestimating the quality of life in the sectors 'Self-esteem', 'Friends' and in the 'Total' score than if the mother answers the questions alone.

The higher the self-assessed quality of life, then the less likely it is that the parents will overestimate the quality of life in all domains (Table 3b). If the parents report behavioural abnormalities of their children, there is a reduced likelihood for each quality of life dimension that the parents overestimate the quality of life. The same applies when parents assess the state of health of their child not as very good, except that in this case the evaluation of the 'Family' scale is not influenced.

Regarding the 'Physical' dimension and the quality of life with respect to Friends, there is a greater likelihood of parental overestimation in the case of boys than girls. With increasing age of the children, the likelihood of parental overestimation of their child's quality of life sinks for the 'School' dimension, whereas the likelihood of overestimating the quality of life with respect to Friends increases with the age of the child. Parents with a migration background are more likely to overestimate the quality of life of their child in the dimensions 'Psychological', 'Family', 'Friends' and in the 'Total' score than parents without a migration background, whereas regarding 'School' there is a lower chance of parental overestimation in migrant families than families without migration background. If mother and father together or a third-person answers the questionnaire as proxy, then there is a greater chance that the 'Psychological' dimension of quality of life will be overestimated.

## Discussion

The purpose of the present paper was to compare self-assessment and proxy assessment by parents of the quality of life of children and adolescents in a representative German survey. In summary we found low to moderate correlations between the values for parents and children. Inmost quality of life dimensions the effect size of the mean difference between parents and children score was moderate. Children's gender, emotional-and behavioural problems, family climate, migration status and parental gender were associated with patterns of disagreement between child and parent scores in most KINDL scales. From our results it can be concluded that boys and migrants and especially boys with migrant status constitute a group at higher risk for parental non-recognition of a decreased Quality of life.

In accordance with the findings of other studies, the agreement between the assessments of parents and their children was relatively small [10,11]. However, the correlation coefficients for the KINDLR in this study and also in the study by Jozefiak et al. [8] were higher than for the PedsQL [9], and they are comparable with the values of KIDSCREEN [15] or TACQOL [7] for healthy children. As in other studies, we found that the agreement for daughters is greater than for sons [15], and for adolescents is greater than for children [8,15] (Tab.2). In contrast, Creemens et al. [9] found greater agreement for children than for adolescents.

There are considerable differences, depending on the dimension of quality of life considered. The greatest agreement (51.8%) was reached for the 'Psychological' scale, although the correlation coefficients (Pearson and ICC) are only about 0.32. As far as differences are concerned, parental underestimation of the quality of life in this dimension was occurred more often than overestimation. Least comparable were parent and child assessment of 'Self-esteem'. Here only about a third of parents agreed with the assessment of their child. The greatest proportion of parents (48.2%) overestimated the quality of life of their child in this dimension. Concerning 'Family', the parental assessment of the quality of life was most often too low. In contrast to other studies [4,8] we did not find the greatest agreement for the 'Physical' scale. A review of the items it contains shows that this KINDL scale also focuses more on subjective perceptions, "... I felt ill", "... I had a headache or tummy-ache", "... I was tired and worn-out" "I felt strong and full of energy". Parents may not have any direct access to these individual insights. The 'Physical' scale of other instruments may ask for more externally visible signs of behaviour which can also be directly observed by the parents. For example most of the PedsQL items ask directly visible activities e.g. "hard to walk more than one block", "hard to run", hard to do sports activities" "hard to lift something heavy" "hard to take bath/shower". "trouble getting along with other teens", "other teens tease", "cannot do things other teens can do", "hard to pay attention in class", "forgot things", "trouble with schoolwork", "miss school". Only the 5 emotional items ("feel afraid", "feel sad", etc) are less visible for parents (as is the case with the KINDL-R items).

As described in other studies [11,13,15], we also found a difference in the extent to which factors influence the agreement or disagreement, depending on the dimension of quality of life considered. The most important influence was the level of the child's self-assessment followed by the parent's assessment of the subjective health, or reported emotional abnormalities. If there were emotional abnormalities and/or the state of health of the child was reported to be moderate, poor or very poor, then the parents tended to underestimate the quality of life of these children. This could be due to the so-called Response-Shift phenomenon. Children with chronic health problems may have developed improved strategies for coping with them. At the same time, these children may also have adapted their internal assessment standards to their state of health and after some time may report a higher quality of life than an observer such as their parents would expect [38,39].

Parents with a lower socio-economic status tended to underestimate the quality of life of their children less frequently with respect to Friends. It goes beyond the scope of this study to consider whether parents in socio-economically disadvantaged families come to accept social disadvantages and limitations as inevitable [40] and are for this reason less likely to underestimate the quality of life of their children with respect to Friends.

Additional analyses reported in a companion paper [16] showed the parent reports were internally more consistent than the children reports. However both versions were found to enable a valid and reliable assessment. From a theoretical point of view and for the sake of presenting a clear argumentation we still would consider the subjective self reports as being more valid than the parental reports.

The study was based on a national representative sample of the general population of children and adolescents in Germany. Thus it is likely that the results are generalizable to specific populations encompassed by our sample. Clearly our findings cannot be generalized to institutionalized child populations, or child populations with strong mental retardation.

It is not possible to conclude how far these results can be generalized to other generic quality of life scales. Even in the case of similar scale and item content the exact wording of a particular item might lead to responder specific response behaviour that cannot be predicted from our findings.

This study is subject to methodological limitations. A basic limitation is that the analysis of a cross-sectional dataset excludes the possibility of a causal interpretation of differences between parents and children. A further limitation lies in the statistical analysis using a categorisation of the differences between parents and children into three classes (overestimation, underestimation, agreement). This means that psychometric information can be lost. As a result our analyses could tend to underestimate the strength of the effects being analysed. On the other hand, our evaluation strategy does make it possible to concentrate on practically important differences between parents and their children. An advantage of the chosen approach is that the direction of parent-child-disagreement can be differentiated, which is part of the key message of our analyses. The role of parental stress was beyond the scope of this investigation. In an additional study module on mental health-the BELLA Study [41], details of parental stress were also considered for a sub-sample.

The strengths of the study lie in the fact that, for the first time, health-related quality of life has been studied in a large sample of 11-17 year old children and adolescents, representative of the entire population in Germany. In particular the explicit consideration of families with a migration background can provide valuable insights.

## Conclusions

KiGGS shows that parental reports cannot adequately replace self-assessment for 11-17 year olds. In view of the different underlying perspectives, the parental assessments should where possible only be regarded as providing supplementary information. Where there is no self-assessment, due to ill-health or cognitive limitations, then the different perspectives represent a problem. Our findings can help with the interpretation of isolated parental assessments.

## Competing interests

The authors declare that they have no competing interests.

## Authors' contributions

UE has conducted the statistical analyses and has conceptualised and written the manuscript. BK and URS were the principal investigators of the study and have advised the writing of the manuscript and have revised the paper. ME has assisted in the statistical analyses and has revised the manuscript. All authors have read and approved the final manuscript.
